# Novel Immunohistochemical Profiling of Small-Cell Lung Cancer: Correlations Between Tumor Subtypes and Immune Microenvironment

**DOI:** 10.3390/diagnostics14232660

**Published:** 2024-11-26

**Authors:** Alon Vigdorovits, Gheorghe-Emilian Olteanu, Andrei-Vasile Pascalau, Radu Pirlog, Ioana Berindan-Neagoe, Ovidiu-Laurean Pop

**Affiliations:** 1 Department of Morphological Disciplines, University of Oradea, 410087 Oradea, Romania; alonvigdorovits@gmail.com (A.V.); pascalau.andrei@gmail.com (A.-V.P.); drovipop@yahoo.com (O.-L.P.); 2 British Columbia Cancer, Department of Pathology, Vancouver, BC V5Z 4E6, Canada; 3 Department of Pathology and Laboratory Medicine, University of British Columbia, Vancouver, BC V6T 1Z7, Canada; 4 Département de Pathologie, Hôpitaux Universitaires Henri Mondor, AP-HP, 94010 Créteil, France; pirlog.radu@yahoo.com; 5 INSERM U955, Université Paris Est Créteil, 94010 Créteil, France; 6 Research Center for Functional Genomics, Biomedicine and Translational Medicine, Iuliu Hatieganu University of Medicine and Pharmacy, 400337 Cluj-Napoca, Romania; ioananeagoe29@gmail.com; 7 Doctoral School, Iuliu Hatieganu University of Medicine and Pharmacy, 400337 Cluj-Napoca, Romania

**Keywords:** SCLC, molecular classification, TILs, tumor immune microenvironment

## Abstract

Background/Objectives: Small-cell lung cancer (SCLC) is a highly aggressive malignancy with an emerging molecular classification based on the expression of the transcription factors ASCL1, NEUROD1, and POU2F3. This study aimed to explore the relationship between these novel subtypes and the tumor immune microenvironment (TIME), particularly CD8+ and CD4+ tumor-infiltrating lymphocytes (TILs). Methods: In 51 cases of patients with SCLC, immunohistochemical (IHC) stains for ASCL1, NEUROD1, POU2F3, CD56, Ki67, CD8, and CD4 were performed. H-scores for the novel transcription factors were calculated to determine tumor subtype. CD8+ and CD4+ TIL counts were averaged across 10 high-power fields. The Kruskal–Wallis test and subsequent post hoc Dunn tests were used to determine the differences in transcription factor expression and TILs across subtypes. Results: In our cohort, 68.62% of our cases were SCLC-A, 9.80% were SCLC-N, 7.84% were SCLC-P, and 13.72% were SCLC-I. Significant differences were observed in the expression of ASCL1, NEUROD1, and POU2F3 across subtypes. CD8+ TILs were more abundant in SCLC-P and SCLC-I. CD8+ TILs were negatively correlated with ASCL1 expression (*p* < 0.05) and positively correlated with POU2F3 expression (*p* < 0.005). Conclusions: This study highlights the need to integrate the novel SCLC classification with data regarding the TIME to better inform patient prognosis and treatment.

## 1. Introduction

Small-cell lung cancer (SCLC) is one of the deadliest malignant tumors [1,2]. SCLC was initially described in 1926 by Barnard as mediastinal *oat cell sarcoma* [3]. In 2020, a total of 232,086 SCLC cases were diagnosed around the world, accounting for 10.51% of all lung cancer cases [4]. According to one study analyzing the SEER database, its incidence has steadily declined from 8.8/100,000 in 2000 to 4.8/100,000 in 2019. The proportional incidence of SCLC relative to NSCLC has also decreased—from 14.5% in 2000 to 11.8% in 2019 [5]. It is characterized by accelerated growth, early metastatic potential, genomic instability, and the quasi-ubiquitous inactivation of *TP53* and *RB1* via biallelic loss [6,7]. SCLC is associated with smoking history in the majority of patients [8]. Less than 2.5% of cases develop in light/never smokers, who have a similarly dismal prognosis [9]. The implementation of screening with low-dose computed tomography (LDCT) has reduced lung cancer-associated mortality; however, most of the mortality reduction has come from non-small-cell lung cancer (NSCLC) [10]. One study explored the Dutch cancer registry and identified that between 1989 and 2020, the proportion of women diagnosed with SCLC increased from 22% to 50% [11].

Due to the high metastatic potential of SCLC, around two-thirds of patients present with tumors outside the chest at diagnosis, greatly reducing the overall benefit offered by curative multimodal therapy [12]. In recent years, targeted therapies directed at oncogenic drivers have greatly improved treatment outcomes in patients with NSCLC; however, because of its vastly different underlying biology, SCLC has yet to reap the benefits of targeted treatment [13]. In the 1980s, platinum-based chemotherapy was shown to improve survival in patients with limited-stage SCLC, while platinum combinations, which included etoposide, improved survival for patients with extensive disease [14]. SCLC tumors are extremely chemosensitive when initiating treatment [15]. Recurrence, however, occurs rapidly, making SCLC very difficult to treat [16]. Despite increased response rates, relapse was also very common, leading to a progression-free survival (PFS) of under 6 months and a median survival of under 9 months [17]. The Dutch cancer registry study found that overall survival (OS) for stage IV SCLC had doubled from 3% to 6% between 1989 and 2020 [11]. Etoposide-platinum combination chemotherapy represented the standard of care until 2019, when association with anti-programmed death-ligand 1 (anti PD-L1) immunotherapy showed improved outcomes, with some patients reaching a survival of 3 years [18,19]. Previously, it was observed that SCLC is associated with genomic instability and thus a high rate of somatic mutations. Studies of the tumor microenvironment (TME) revealed an enrichment for effector T cells, which indicated the potential to support an anti-tumor response with checkpoint inhibition [20].

SCLC was classically considered as a single disease entity with neuroendocrine (NE) features. However, it was observed that some cases have a focal, weak, or even absent expression of NE markers [21]. The extent of the heterogeneity of SCLC has only recently been explored. Initially, preclinical studies based on SCLC cell lines, mouse models, and patient-derived xenografts revealed distinct subtypes of SCLC characterized by gene expression programs driven by the transcription factors achaete-scute homolog 1 (*ASCL1*), neurogenic differentiation factor 1 (*NEUROD1*), POU class 2 homeobox 3 (*POU2F3*), and yes-associated protein 1 (*YAP1*) [22]. A high expression of NE markers was shown to be associated with the expression of either *ASCL1* or *NEUROD1* [23]. Following these findings, a proposal emerged, suggesting subtyping SCLC into four subtypes defined by the RNA expression of *ASCL1*, *NEUROD1*, *POU2F3*, and *YAP1*, called SCLC-A, SCLC-N, SCLC-P, and SCLC-Y [22]. A study by Baine et al. was the first study that used surrogate immunohistochemical (IHC) analysis for novel SCLC subtypes, in which they confirmed the previous findings in patient samples, while also documenting a significant level of co-expression between ASCL1 and NEUROD1. They also showed that POU2F3 was associated with a low-expressing NE phenotype, with its expression being mutually exclusive with that of ASCL1 and NEUROD1—a finding confirmed in a subsequent study [24]. Baine et al. did not find evidence that YAP1 defines a distinct subgroup of SCLC, as previously thought. Gay et al. proposed the SCLC-inflamed (SCLC-I) subtype as the fourth subtype, replacing SCLC-Y. SCLC-I had a unique inflamed gene expression profile, with an increased expression of CD8A and CD8B, suggesting cytotoxic T cell infiltration, as well as a high expression of *CD274*, which encodes for Programmed Death Ligand 1 (PD-L1), and *PDCD1* [25]. Single-cell sequencing has revealed that while *ASCL1*, *NEUROD1*, and *POU2F3* were exclusively expressed in malignant cells in SCLC, *YAP1* was more frequently expressed in normal epithelial cells [26]. One recent study has found that YAP1-positive cases had a higher medial OS (35.6 months) compared to YAP1-negative cases (16.9 months), as determined via IHC. The authors suggested that YAP1 might be more suited as a favorable prognostic indicator rather than a dominant subtype marker [27]. Another study found that pathogenic mutations in *SMARCA4* were present in six of eight SCLC-Y cell lines, and were correlated with reduced SMARCA4 mRNA. Pathologists reviewed these tumors and observed features consistent with thoracic SMARCA4-deficient undifferentiated tumors [28].

These novel subtypes may provide avenues for targeted treatment in SCLC. In SCLC-A, it was observed that ASCL1 expression is correlated with a high BCL-2 and DLL3 expression [29,30,31]. *MYC* activation was observed in the SCLC-N subtype, indicating potential therapeutic benefit from Aurora kinase inhibitors, arginine deprivation, and mTOR inhibition [32,33]. In SCLC-P, PARP (poly ADP ribose polymerase) inhibitors, IGF-1R inhibitors, and nucleoside analogs appear to be potential treatment options [34,35]. SCLC-I has the potential of responding to immune checkpoint inhibition, due to the inflamed gene expression profile [25]. Shirasawa et al. investigated the association between CD8+ tumor-infiltrating lymphocytes (TILs) and SCLC subtypes, and found that SCLC-I tumors were characterized by a high TIL count [36]. Another study found no correlation between CD8+ TILs and SCLC subtypes [37]. Chiang et al. showed that patients with SCLC-A and SCLC-N had a similar PFS compared to those with non-SCLC/A after treatment with immune checkpoint inhibitors plus chemotherapy. They also observed that approximately 50% of patients went through a subtype switch after disease progression. Patients with transformed SCLC showed a significantly worse PFS than those with newly diagnosed SCLC after chemoimmunotherapy [38].

The aim of our study was to explore the variation in transcription factor expression defining the novel SCLC subtypes using IHC. We also sought to investigate the correlation between transcription factor expression and the tumor immune microenvironment (TIME) by evaluating CD8+ and CD4+ tumor-infiltrating lymphocytes (TILs). By identifying patterns in subtype-specific or transcription factor-dependent associations, this study attempts to provide insights that could potentially inform diagnostic and therapeutic strategies in the future.

## 2. Materials and Methods

### 2.1. Case Selection

Cases included in the study were represented by the biopsies of 51 patients that were diagnosed with SCLC in our institution between November 2021 and January 2024. Cases were reviewed by a thoracic pathologist (O.L.P. and G.E.O.) before being included in the analysis. This study was performed in accordance with the Declaration of Helsinki and was approved by the Ethics Committee of Bihor County Clinical Emergency Hospital (nr. 8332/15.03.2024).

### 2.2. Immunohistochemistry

IHC stains were performed for ASCL1 (clone ESS4Q, 1:300 dilution, Cell Signaling Technology, Beverly, MA, USA), NEUROD1 (clone D90G12, 1:50 dilution, Cell Signaling Technologies), POU2F3 (E5N2D, 1:200 dilution, Cell Signaling Technologies), CD56 (clone 123C3, ready-to-use, Agilent, Santa Clara, CA, USA), Ki67 (clone MIB-1, ready-to-use, Agilent), CD8 (clone C8/144B, ready-to-use, Agilent), and CD4 (clone 4B12, ready-to-use, Agilent) using the Dako Autostainer Link 48 platform (Agilent). Two thoracic pathologists (O.L.P. and G.E.O.) performed the scoring for each marker. For ASCL1, NEUROD1, POU2F3, and CD56, a histoscore (H-score) was calculated by multiplying an intensity score (1 = weak, 2 = moderate, 3 = strong) with the percentage of positive cells (1–100%) [39]. The Ki67 index was measured as the percentage of positive cells. CD8+ and CD4+ TILs for each case were determined by averaging across 10 random high-power fields (HPFs). The SCLC subtype was determined based on the highest H-score between ASCL1, NEUROD1, and POU2F3. Expression was considered significant at an H-score of at least 50. Double expression was defined as at least two markers with a minimum H-score of 50. In cases with combined histology, the H-score was calculated only in the SCLC component.

### 2.3. TIL Assessment Methodology and Specimen-Specific Considerations

As stated, CD8+ and CD4+ TILs were evaluated in 10 random HPFs (400× magnification); these fields, even if random, were selected systematically, i.e., areas with good preservation and minimal crushing artifacts, and included both intratumoral and stromal-interface regions, avoiding areas of necrosis or technical artifacts. For combined histology cases, only SCLC components were evaluated. Regarding the specimen-specific considerations, i.e., for biopsies (96% of cases), all available tumor area was assessed—a minimum of 100 viable tumor cells were required for scoring—and the areas with crushing artifacts were excluded. For FNA specimens (4% of cases), cell block sections were evaluated, and the same scoring criteria was applied as for biopsies; adequate cellularity was confirmed before scoring.

### 2.4. Statistical Analysis

Shapiro–Wilk normality testing was performed for all numerical variables used in the analysis. Principal component analysis (PCA) was performed in order to better visualize and explore the dataset and the relationship between subtypes. The Kruskal–Wallis test was used to compare the differences across SCLC subtypes in ASCL1, NEUROD1, POU2F3, and CD56 expression; the Ki67 index; and CD8+ and CD4+ TILs/HPFs. Dunn’s multiple comparison test was performed for post hoc pairwise comparisons after a Kruskal–Wallis test identified a significant difference between groups. Spearman’s rank correlation coefficient was calculated for all numerical variables analyzed. GraphPad Prism 9 was used for statistical evaluation and plot generation.

## 3. Results

### 3.1. Patient and Tumor Characteristics

**Figure 1:**
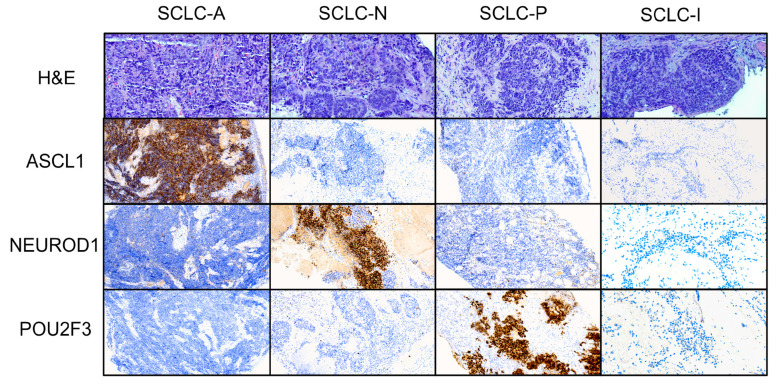
SCLC subtypes—H&E, and transcription factor IHC staining—at 200× magnification.

Out of the 51 patients, 7 (13.72%) were female and 44 were male (86.28%). The mean age was 64.90 years (SD = 9.70). Available specimens comprised 49 biopsies (96%) and 2 fine-needle aspirates (4%). One biopsy was from a metastatic liver lesion. Histologically, 46 of the cases (90.2%) were pure SCLC, 4 (7.84%) were combinations of SCLC and squamous cell carcinoma, and 1 case (1.96%) was a combination with adenocarcinoma. Examples of images from each subtype can be seen in Figure 1.

With respect to subtype distribution, 35 cases (68.62%) were classified as SCLC-A, 5 (9.80%) were classified as SCLC-N, 4 (7.84%) were classified as SCLC-P, and 7 (13.72%) were classified as SCLC-I. Double ASCL1/NEUROD1 expression was present in five cases (9.8%). One case (1.96%) had an expression of both ASCL1 and POU2F3, but this expression was present in different regions of the tumor and did not overlap.

### 3.2. Statistical Analysis

**Figure 2:**
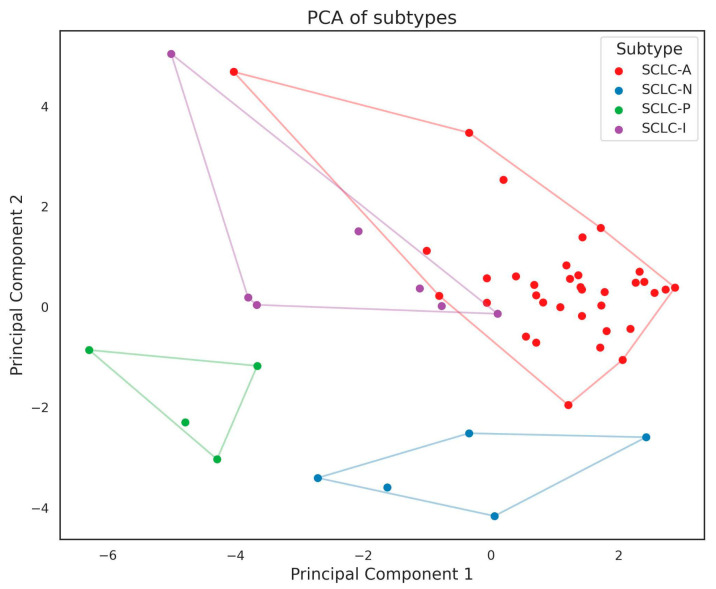
Subtype principal component analysis (PCA).

PCA showed distinct clusters for SCLC-N and SCLC-P, while SCLC-A and SCLC-I had an overlap of the convex hulls (Figure 2). The mean and standard deviation of ASCL1, NEUROD1, and POU2F3 H-scores for each SCLC subtype are highlighted in Table 1.

**Figure 3:**
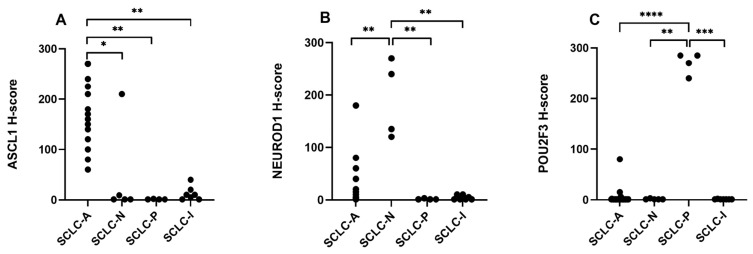
Differences in expression levels for ASCL1 (**A**), NEUROD1 (**B**), and POU2F3 (**C**) across subtypes. * *p* < 0.05; ** *p* < 0.005; *** *p* < 0.0005; **** *p* < 0.00005.

ASCL1, NEUROD1, and POU2F3 H-scores were not normally distributed according to the Shapiro–Wilk test. The Kruskal–Wallis test performed for the ASCL1 H-score revealed a significant difference across subtypes (*p* < 0.0001). Dunn’s multiple comparison test was significant for SCLC-A vs. SCLC-I (*p* = 0.001), SCLC-A vs. SCLC-N (*p* = 0.018), and SCLC-A vs. SCLC-P (*p* = 0.002). When comparing NEUROD1 H-scores across SCLC subtypes, the Kruskal–Wallis test was significant (*p* = 0.001), with Dunn’s test highlighting significant pairwise differences for SCLC-N vs. SCLC-A (*p* = 0.003), SCLC-N vs. SCLC-P (*p* = 0.002), and SCLC-N vs. SCLC-I (*p* = 0.004). For the POU2F3 H-score, we observed a significant difference between subtypes (*p* < 0.0001), while Dunn’s multiple comparison test revealed significant variation for SCLC-P vs. SCLC-A (*p* < 0.0001), SCLC-P vs. SCLC-N (*p* = 0.003), and SCLC-P vs. SCLC-I (*p* = 0.0005). These differences are illustrated in Figure 3.

**Figure 4:**
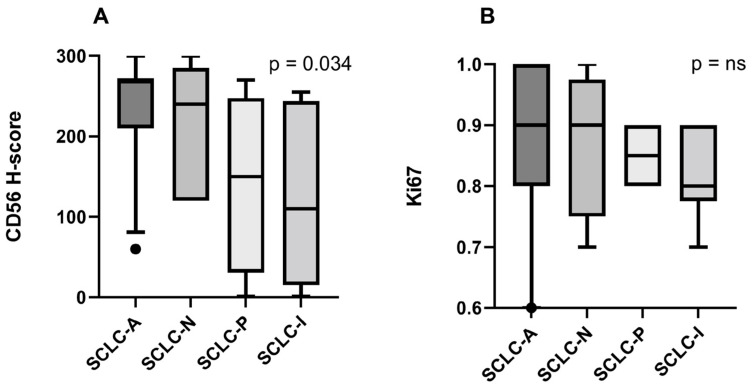
CD56 H-score (**A**) and Ki67 (**B**) across subtypes. Ns = not significant.

CD56 H-score and Ki67 were not normally distributed according to the Shapiro–Wilk test. The following CD56 H-scores were found—SCLC-A: 234.5 (SD = 60.61); SCLC-N: 210 (SD = 84.85); SCLC-P: 142.8 (SD = 112.8); and SCLC-I: 122.7 (SD = 115.5). The Kruskal–Wallis test was statistically significant with regard to differences in CD56 H-score across SCLC types (*p* = 0.034). However, Dunn’s multiple comparison test did not highlight any significant pairwise differences (*p*-values > 0.05). The calculated mean Ki67 indexes were as follows—SCLC-A: 85.63% (SD = 13.43%); SCLC-N: 87.5% (SD = 12.58%); SCLC-P: 85% (SD = 5.77%); and SCLC-I: 81.67% (SD = 7.52%). No significant differences were observed for Ki67 across SCLC types (Figure 4).

**Figure 5:**
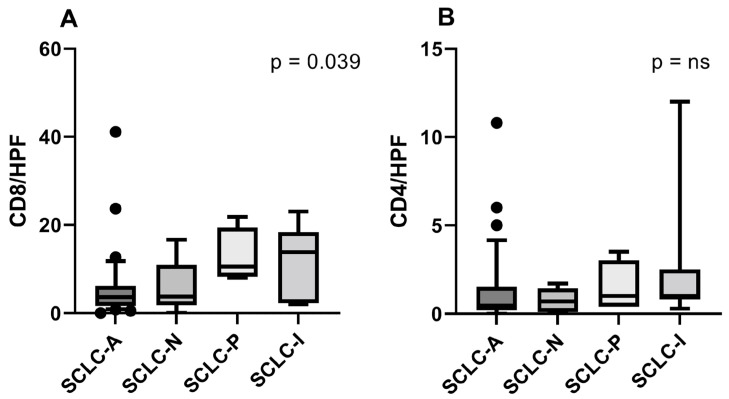
CD8+ lymphocytes/HPFs (**A**) and CD4+ lymphocytes/HPFs (**B**) across subtypes. Ns = not significant.

TILs/HPFs were not normally distributed according to the Shapiro–Wilk test. Calculated average CD8+ TILs/HPFs were as follows—SCLC-A: 5.56 (SD = 7.82); SCLC-N: 5.82 (SD = 6.329); SCLC-P: 12.7 (SD = 6.3); and SCLC-I: 11.31 (SD = 8.37). The number of CD8+ cells per HPF count were statistically significant between SCLC types (*p* = 0.039). Pairwise comparisons using Dunn’s test did not reveal any significant differences. The computed mean CD4+ TILs/HPFs were as follows—SCLC-A: 1.32 (SD = 2.17); SCLC-N: 0.76 (SD = 0.70); SCLC-P: 1.47 (SD = 1.46); and SCLC-I: 2.7 (SD = 4.15). CD4+ TILs/HPFs numbers did not present significant differences across subtypes (Figure 5).

**Figure 6:**
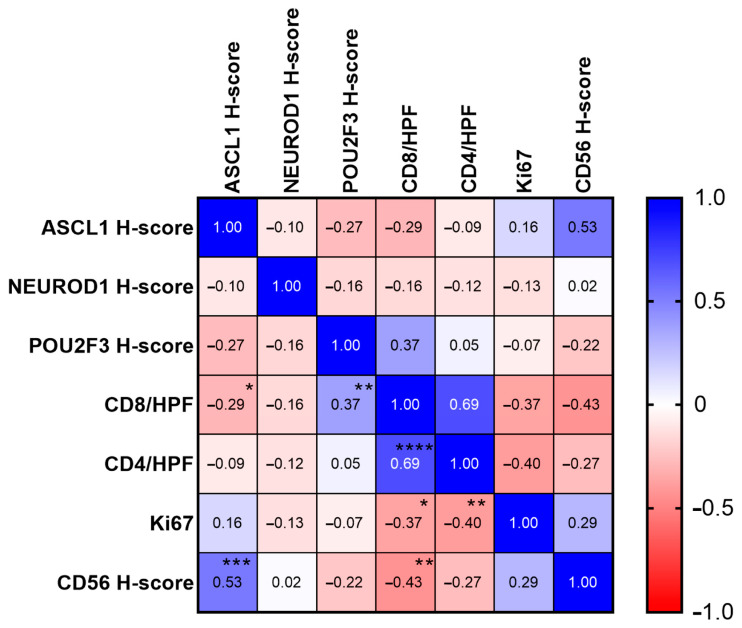
Correlation matrix. Spearman’s ρ ranging from -1.0 to 1.0 shown for each pair, alongside clinical significance (* *p* < 0.05; ** *p* < 0.005; *** *p* < 0.0005; **** *p* < 0.00005).

Spearman’s correlation analysis (Figure 6) revealed a statistically significant positive correlation between CD8+ TILs/HPFs and POU2F3 H-score (r = 0.37, *p* = 0.008), CD56 H-score and ASCL1 H-score (r = 0.53, *p* < 0.001), and CD4+ TILs/HPFs and CD8+ TILs/HPFs (r = 0.69, *p* < 0.0001). Significant negative correlations were identified between CD8+ TILs/HPFs and ASCL1 H-score (r = −0.29, *p* = 0.042), CD8+ TILs/HPFs and Ki67 (r = −0.37, *p* = 0.014), CD4+ TILs/HPFs and Ki67 (r = −0.40, *p* = 0.007), and CD8+ TILs/HPFs and CD56 H-score (r = −0.43, *p* = 0.003).

## 4. Discussion

In this study, we analyzed the expression of ASCL1, NEUROD1, and POU2F3, as well as their association with the TIME, in relationship to the novel SCLC subtypes. In our cohort, we report a subtype distribution of 68.62% SCLC-A, 9.80% SCLC-N, 7.84% SCLC-P, and 13.72% SCLC-I, which is similar to that reported in previous studies, with SCLC-A being the most common subtype [24,37]. The co-expression of ASCL1 and NEUROD1 was reported in five cases, while co-expression between ASCL1 and POU2F3 was seen in one case, in which the expression of the two markers was mutually exclusive, being present in different cell populations. Baine et al. also reported mutually exclusive expressions of ASCL1 and POU2F3 [40]. This could suggest the existence of different subclones within these tumors. Further research should also explore the possibility that cases with co-expression have a distinct prognosis. We found significant differences in CD56 expression between subtypes, in keeping with the evidence that SCLC-A and SCLC-N have a higher expression of NE markers [24,41]. We also found that SCLC-I tumors tend to have a slightly lower Ki67 index, without reaching formal statistical significance.

CD8+ TIL/HPF counts revealed significant differences across subtypes, with SCLC-P and SCLC-I showing increased CD8+ TILs compared to SCLC-A and SCLC-N. CD4+ TILs/HPFs did not reveal significant differences between the subtypes. However, CD4+ and CD8+ TILs showed a significant positive correlation across all subtypes (r = 0.69, *p* < 0.0001) with higher CD4+ TILs observed in the SCLC-P and SCLC-I subtypes. Even though there were no significant pairwise differences in CD8+ TILs, they were significantly negatively correlated with ASCL1 H-score (r = −0.29, *p* = 0.042), which highlights the general trend in cases with ASCL1-dominant expression to have less immune cell infiltration, a finding described in other studies [36]. We also identified a statistically significant positive correlation between POU2F3 H-score and CD8+ TILs/HPFs (r = 0.37, *p* = 0.008). Ki67 was negatively correlated with both CD8+ (r = −0.37, *p* = 0.014) and CD4+ TILs/HPFs (r = −0.40, *p* = 0.007).

Previous studies have suggested that subtypes determined using IHC and subtyping using transcriptomic data do not completely overlap [36]. One proteogenomic study reported that out of 3764 genes, 89% showed positive correlation between mRNA and protein levels, but only 32% had a statistically significant correlation [42]. On the other hand, Park et al. showed a high concordance of IHC-based subtyping with whole-transcriptome sequencing and non-negative matrix factorization clustering methods, while also observing that inflamed tumors were more likely to benefit from first-line immunotherapy compared to the non-inflamed phenotype. They observed the inflamed phenotype in 26% of IHC-based SCLC-A, 11% of SCLC-N, and 53% of SCLC-P [43]. These findings further emphasize the need to assess the TIME in all subtypes to identify patients that would benefit from immunotherapy. Shirasawa et al. noted that SCLC-A determined using IHC consisted of all four transcriptional subtypes, with some patients having durable responses to immunotherapy [36]. Our cohort contains three outliers classified as SCLC-A that showed increased CD8+ lymphocytes, which is suggestive of a possible inflamed transcriptomic profile. These cases also showed lower Ki67 proliferation indexes. POU2F3 expression was associated with increased CD8+ TILs. In a subset analysis of the Impower 133 study, it was shown that patients with SCLC-P had the shortest median survival out of the four subtypes [25]. SCLC-P tumors frequently present with *MYC* amplification, which could increase their resistance to cytotoxic CD8+ TILs via PD-L1 and CD47 [44]. Another issue regarding the difference between IHC-determined and transcriptomics-defined subtypes is the stability under treatment. One study showed that the IHC-determined subtype was stable after chemotherapy but highlighted the need for studies with paired RNA expression data [45]. These observations highlight the potential importance of evaluating TILs prospectively as a possible cross-subtype predictor for immunotherapy response.

Our study has certain limitations. The sample size of 51 patients could limit the generalizability of our findings. Even though IHC provides valuable insight into the expression of the novel transcription factors, having access to RNA expression data would greatly increase insight into our findings. Proteomic profiling could also aid in bridging the gap between transcriptomic studies and IHC subtyping, as well as offering insight into the biology of SCLC. The high variability in protein expression as well as cancer heterogeneity are the main challenges faced by proteomics-based approaches [46]. Computational pathology is another tool that has been widely used in multiple diagnostic, prognostic, and predictive use cases [47,48,49]. In the future, computational approaches could be used in conjunction with genomic, transcriptomic and proteomic data to predict the response of SCLC patients to immunotherapy or targeted therapy, increasing the granularity of predictions offered by data such as TILs and IHC subtypes.

In conclusion, this study helps reinforce the novel classification of SCLC into molecular subtypes governed by the transcription factors ASCL1, NEUROD1, and POU2F3, while also providing context with respect to the TIME, further growing the body of literature with the aim of providing patients with better diagnostic, prognostic, and therapeutic options in the face of this highly aggressive malignancy.

## Figures and Tables

**Table 1:** Mean and standard deviation of ASCL-1, NEUROD1, and POU2F3 H-scores.

Subtype	ASCL1 H-Score	NEUROD1 H-Score	POU2F3 H-Score
SCLC-A	187 (SD = 63.41)	18.46 (SD = 35.57)	3.82 (SD = 13.49)
SCLC-N	44.40 (SD = 92.64)	207 (SD = 73.79)	1.4 (SD = 0.89)
SCLC-P	1.25 (SD = 0.5)	1.5 (SD = 1)	270 (SD = 21.21)
SCLC-I	12.43 (SD = 13.82)	4.143 (SD = 4.25)	1.14 (SD = 0.37)

## Data Availability

The data used in this study can be made available upon reasonable request.
